# Amyand’s hernia with appendicitis in a 64-year-old male: a case report

**DOI:** 10.1093/jscr/rjaf205

**Published:** 2025-04-25

**Authors:** Jorge A García Garza, Homero Zapata Chavira

**Affiliations:** General Surgery Service of Hospital Regional de Monterrey Issste, Monterrey C.P. 64380, Mexico; General Surgery Service of Hospital Regional de Monterrey Issste, Monterrey C.P. 64380, Mexico

**Keywords:** Amyand’s hernia, inguinal hernia, appendicitis

## Abstract

We present a medical case of a 64-year-old male patient who exhibited symptoms of a hernia in the right inguinoscrotal region, which could not be reduced, and was accompanied by a systemic inflammatory response, ultimately revealing a perforated appendicitis during surgery as the hernia’s contents. The relatively rare occurrence and lower probability of an Amyand’s hernia may prompt a question about the most effective surgical treatment for our patient. The main aim of this study is to expand the current body of research on this condition, which should allow for a more thorough understanding of it.

## Introduction

Amyand’s hernia, described for the first time in 1735 by Dr. Claudius Amyand, is a low-frequency pathology with a prevalence of 1% of all inguinal hernias and up to 0.1% of all cases of appendicitis. It affects men more than women. The rate of occurrence increases at both ends of the lifespan and begins to decline following childhood, only to reach a second peak in incidence after 70 years of age. The management of this pathology must be individualized for each case and may or may not opt ​​for an appendectomy depending on the conditions of the appendix. However, appendectomy plus repair of the hernia defect without mesh is considered correct in cases with suppurative appendicitis [1–3]. We present the following case of a 64-year-old man with the incidental discovery of an Amyand hernia during the intraoperative period of an incarcerated indirect inguinal hernia.

## Case report

A 64-year-old male patient with a 10-year history of systemic arterial hypertension and a surgical history of umbilical hernioplasty 18 years ago is being presented. He comes to the emergency room with inguinodynia 8/10 on the visual analog scale, erythema, increased temperature, and non-reducible swelling of the right scrotal sac, which has worsened over the past 24 h before admission, along with diffuse abdominal pain without signs of peritoneal irritation but with suggestive signs of intestinal occlusion.

**Figure 1:**
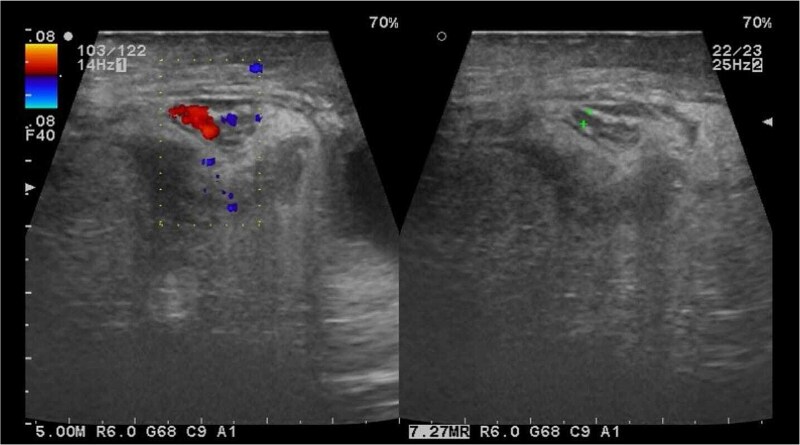
Testicular ultrasound with intestinal loops.

Paraclinical studies revealed leukocytosis of 18 thousand at the expense of >85% neutrophils. Ultrasound of the inguinal region revealed a right testicle of normal dimensions with increased peritesticular fluid, a hernial ring of 39 × 28 mm with passage of intestinal loops, mesentery, and vascular structures which is not reducible during the examination (Fig. 1).

The diagnosis of an incarcerated inguinal hernia is confirmed using clinical, laboratory, and imaging data, computed tomography scan was avoided to not delayed the surgical treatment, which was perform immediately.

An inguinal approach was chosen, which led to the identification of an indirect inguinal hernia. The hernia content is reduced and the hernial sac is partially removed because of adherences in the sac fundus. Upon dissecting the hernial sac, a visible discharge of pus is seen near the spermatic cord, and a 1 cm opening or perforation is identified at the sac fundus. Throughout the inguinal canal, thorough antiseptic irrigation is carried out alongside a complete removal of the hernial sac. A Bassini’s technique for the hernioplasty, and the source of the purulent material was investigated through an abdominal procedure.

**Figure 2:**
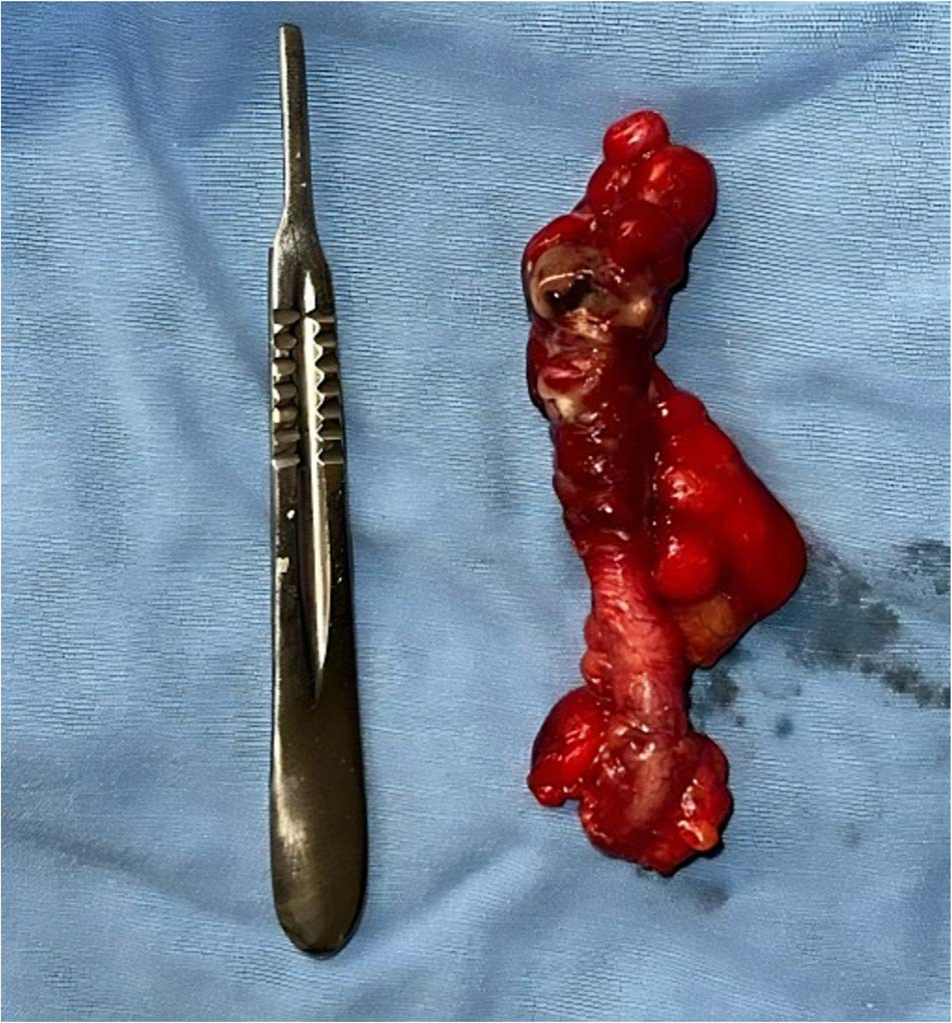
Appendectomy grade IV; perforation data, comparison with No. 3 scalpel.

We opted not to begin with a laparoscopic exploration due to a high risk of abdominal contamination, and since the initial procedure was an open hernioplasty, an exploratory laparotomy was instead performed, which confirmed that the appendix was swollen, necrotic, and perforated at its tip, with the release of pus, along with a thrombosed artery (grade IV appendicitis) (Fig. 2). The surgery involved the Pouchet technique for the appendectomy plus invagination of the proximal stump. A Penrose drain was placed in the inferolateral region of the affected scrotum to control any remaining pus and localized swelling. The patient went through the post-surgery period without any issues and was monitored for 5 days to ensure a complete antibiotic course. A right scrotal ultrasound examination revealed no evidence of testicular involvement, and the patient was discharged from the hospital.

## Discussion

Amyand’s hernia is a rare pathology and in most cases it is an incidental finding during surgical procedures as occurred in our case, however it can also be found during elective surgeries. Our patient is approaching the second age peak with the highest incidence rate, and it is noted that at this age peak, the likelihood of incarcerated hernia is significantly increased [2–4].

Symptoms typically manifest in the initial 24 h among adults and are often associated with an incarcerated hernia. Leukocytosis is often present, and in our case, ultrasound results indicated the presence of intestinal material within the herniated contents. However, due to the severity of symptoms and high probability of short-term complications, it was decided to perform emergency surgery. In the classification and staging of Losanoff and Basson there are four types; a type I of Amyand’s hernias, accounting a 47% of the cases, involves a normal appendix found during an elective hernioplasty, the debate surrounding whether to remove the appendix during surgery lacks of a definitive answer in the medical literature, and the decision is often based on the surgeons professional judgment and consideration of the patients characteristics and needs. Younger patients are more likely to develop acute appendicitis compared to older patients, the use of prosthetic mesh may be used when needed. Type II is seen in 42% of cases and involves an appendix with signs of inflammatory or septic changes located inside the hernia sac, removal of the appendix is required, although in these cases the use of prosthetic mesh for the hernia repair may have and increased risk of infection. Type III of Amyand’s hernias have a 9% rate, and in these cases, the purulent material spreads beyond the hernia sac, potentially leading to more severe clinical outcomes that may require significant surgical interventions like exploratory laparotomy or orchiectomy; in critically ill patients, hernioplasty may need to be postponed. Type IV hernias account for 2% of cases, and they are characterized by the presence of any Amyand’s hernia complicated by additional pathology outside the hernia sac. Our patient presented a type III vermiform appendix, ⁓9% of the Amyand hernias, since the purulent material had come out of the hernial sac and required profuse lavage of the scrotal region in addition to an exploratory laparotomy to control possible damage; however, no data of peritonitis were found [3, 5].

The treatment was based on the presence of pus outside the peritoneal cavity. Appendectomy was performed due to the presence of suppurative appendicitis, and no mesh was used due to a high risk of infection. A Penrose drain was placed in the scrotum because the sac had perforated, releasing a small amount of purulent material, and there was a high risk of infection [6].

## Conclusion

Amyand’s hernia is often identified incidentally during surgical procedures, and available data in medical literature is limited due to the relatively low occurrence of this pathology. This case is documented to expand our understanding of the underlying pathology and to enhance the existing literature, ultimately facilitating better comprehension and treatment of this condition while also minimizing potential future complications.
